# Three models that predict the efficacy of immunotherapy in Chinese patients with advanced non‐small cell lung cancer

**DOI:** 10.1002/cam4.4171

**Published:** 2021-08-13

**Authors:** Qian Zhao, Butuo Li, Yiyue Xu, Shijiang Wang, Bing Zou, Jinming Yu, Linlin Wang

**Affiliations:** ^1^ Cheeloo College of Medicine Shandong University Jinan China; ^2^ Department of Radiation Oncology Shandong Cancer Hospital and Institute (Shandong Cancer Hospital Shandong First Medical University and Shandong Academy of Medical Sciences Jinan China

**Keywords:** immunotherapy, non‐small cell lung cancer (NSCLC), predictive, score

## Abstract

**Background:**

Many tools have been developed to predict the efficacy of immunotherapy, such as lung immune prognostic index (LIPI), EPSILoN [Eastern Cooperative Oncology Group performance status (ECOG PS), smoking, liver metastases, lactate dehydrogenase (LDH), neutrophil‐to‐lymphocyte ratio (NLR)], and modified lung immune predictive index (mLIPI) scores. The aim of this study was to determine the ability of three predictive scores to predict the outcomes in Chinese advanced non‐small cell lung cancer (aNSCLC) patients treated with immune checkpoint inhibitors (ICIs).

**Methods:**

We retrospectively analyzed 429 patients with aNSCLC treated with ICIs at our institution. The predictive ability of these models was evaluated using area under the curve (AUC) in receiver operating characteristic curve (ROC) analysis. Calibration was assessed using the Hosmer–Lemeshow test (H–L test) and Spearman's correlation coefficient. Progression‐free survival (PFS) and overall survival (OS) curves were generated using the Kaplan–Meier method.

**Results:**

The AUC values of LIPI, mLIPI, and EPSILoN scores predicting PFS at 6 months were 0.642 [95% confidence interval (CI):0.590–0.694], 0.720 (95% CI: 0.675–0.762), and 0.633 (95% CI: 0.585–0.679), respectively (*p* < 0.001 for all models). The AUC values of LIPI, mLIPI, and EPSILON scores predicting objective response rate (ORR) were 0.606 (95% CI: 0.546–0.665), 0.683 (95% CI: 0.637–0.727), and 0.666 (95% CI: 0.620–0.711), respectively (*p* < 0.001 for all models). The C‐indexes of LIPI, mLIPI, and EPSILoN scores for PFS were 0.627 (95% CI 0.611–6.643), 0.677 (95% CI 0.652–0.682), and 0.631 (95% CI 0.617–0.645), respectively.

**Conclusions:**

As mLIPI scores had the highest accuracy when used to predict the outcomes in Chinese aNSCLC patients, this tool could be used to guide clinical immunotherapy decision‐making.

## Graphical abstract

Many tools have been developed to predict the efficacy of immunotherapy, such as lung immune prognostic index (LIPI), EPSILoN [Eastern Cooperative Oncology Group performance status (ECOG PS), smoking, liver metastases, lactate dehydrogenase (LDH), neutrophil‐to‐lymphocyte ratio (NLR)], and modified lung immune predictive index (mLIPI) scores. The aim of this study was to determine the ability of to predict outcomes in Chinese aNSCLC patients treated with immune checkpoint inhibitors (ICIs).

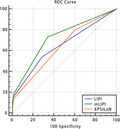

## 1 INTRODUCTION

Over the past decade, the emergence of immunotherapy has significantly changed the therapeutic landscape of advanced non‐small cell lung cancer (aNSCLC).^1^ Nivolumab, a fully human IgG4 monoclonal antibody that targets PD‐1, was initially approved as an immune checkpoint inhibitor (ICI) by the United States Food and Drug Administration (FDA) to treat patients with pretreated aNSCLC.^2^, ^3^, ^4^ Due to a significant improvement in the overall survival (OS) of patients treated with nivolumab compared with standard chemotherapy, pembrolizumab and atezolizumab were then also approved to treat aNSCLC patients.^5^, ^6^, ^7^ Recently, anti‐PD‐1/PD‐L1 agents, combined with platinum‐doublet chemotherapy and bevacizumab, have been used as first‐line aNSCLC treatment and have demonstrated greater survival benefit compared with standard chemotherapy. On 15 June 2018, nivolumab became the first immunologic drug approved by the China Food and Drug Administration (CFDA) to enter the Chinese market. This provided new treatment options for Chinese aNSCLC patients. Following this, pembrolizumab, sintilimab, toripalimab, and other ICIs have been successively launched in China, these drugs enabling many patients to achieve long‐term survival. Despite these survival improvements, however, only a proportion of patients respond to immunotherapy and less than 20% experience durable clinical benefits.^8^, ^9^ It is crucial to identify predictive and prognostic biomarkers to identify patients who are most likely to respond to immunotherapy. PD‐L1 is the only approved and thoroughly explored predictive biomarker to date. However, of the immunologic drugs used to treat NSCLC, only the application of pembrolizumab was related to the expression of PD‐L1. It has been reported that 11%–20% of patients who are PD‐L1 negative also respond to immunotherapy,^10^, ^11^ rendering the predictive role of PD‐L1 insufficient. While more potential biomarkers are currently being explored, such as microsatellite instability‐high (MSI‐H), tumor mutational burden (TMB), and immunogenic signatures, these biomarkers are expensive and none have been verified as playing a vital role in NSCLC.^12^

Peripheral blood inflammatory parameters, such as neutrophil‐to‐lymphocyte ratio (NLR), derived neutrophil‐to‐lymphocyte ratio (dNLR), lactate dehydrogenase (LDH), and platelet‐to‐lymphocyte ratio (PLR), have been shown to have prognostic and predictive values in various malignancies treated with immunotherapy, including NSCLC.^13^, ^14^, ^15^, ^16^, ^17^ In contrast with PD‐L1 and TMB, these peripheral hematology indicators are readily accessible and essentially non‐invasive to measure. Considering the complex organismal inflammatory environment of cancer patients, clinical outcomes should not be influenced by univariate factors only, but more likely by the combination of multiple clinical features and laboratory parameters in patients with aNSCLC. Thus, a scoring model consisting of multiple clinical parameters could perhaps better predict immunotherapy prognosis. In 2018, Mezquita et al. described a lung immune prognostic index (LIPI) based on pre‐treatment blood levels of dNLR and LDH, and these researchers confirmed that LIPI scores are correlated with the prognosis of aNSCLC when treated with ICIs.^18^ Prelaj et al. has since proposed EPSILoN (Eastern Cooperative Oncology Group performance status (ECOG‐PS), smoking, liver metastases, LDH, and NLR) scores, and Moor et al. has proposed a modified lung immune prognostic index (mLIPI) score based on clinical peripheral blood indicators; these scores have been found to predict the efficacy of aNSCLC immunotherapy.^19^, ^20^, ^21^ However, these three models were developed based on Western populations. Due to ethnic, cultural, and economic differences, the prognostic value of these three models for Chinese NSCLC patients requires further verification. In this study, we used the area under the curve (AUC) in the receiver operating characteristic curve (ROC) analysis to determine the ability of LIPI, EPSILoN, and mLIPI scores to predict the outcomes of Chinese aNSCLC patients treated with ICIs.

## 2 METHODS

### 2.1 Study population

Between September 2018 and February 2020, we retrospectively enrolled 429 patients with histologically proven aNSCLC (IIIB–IV) treated with ICIs at the Department of Radiation Oncology, Shandong Cancer Hospital. We reviewed the electronic medical records of these patients and collected baseline clinical, pathological, blood biochemical, sensitive gene mutation status, and PD‐L1 expression data collected prior to ICI therapy. Patients were excluded if they had autoimmune disease, adrenal insufficiency, pulmonary interstitial disease, or systemic immunosuppression. This study was approved by the Ethics Committee of the Department of Radiation Oncology, Shandong Cancer Hospital. Due to the retrospective study design, formal consent was not required. All procedures performed in this study involving human participants were in accordance with the Declaration of Helsinki (as revised in 2013).

Tumor response was assessed by computed tomography (CT) every 8–12 weeks. The performance status of patients was determined by ECOG scores and response to treatment was evaluated by the Response Evaluation Criteria in Solid Tumors (RECIST) guidelines (version 1.1). Objective response rate (ORR) was defined as the proportion of enrolled patients who achieved a complete response (CR) or partial response (PR). Progression‐free survival (PFS) was defined as the time from the commencement of ICI treatment until disease progression or death. OS was defined as the time from the commencement of ICI treatment until death by any cause or final follow‐up.

### 2.2 Calculation of scores

Variable, data source, and cutoff value selection for LIPI, EPSILoN, and mLIPI scores were performed in line with previously published studies^18^, ^19^, ^20^ and clinical practice in China. The LIPI score was the first to be proposed, incorporating only two clinical parameters, dNLR and LDH, and classifying patients into good, intermediate, and poor prognosis subgroups based on the following cutoff values: dNLR≤3 and LDH≤ULN, dNLR>3 or LDH>ULN, dNLR>3 and LDH>ULN. The mLIPI score was a modification of the LIPI score with the addition of the ECOG score to the two clinical parameters, LDH and NLR (instead of dNLR), with a cutoff value of 1.5*ULN for LDH and a cutoff value of 3 for NLR. The EPSILoN score was based on the inclusion of two risk factors, liver metastasis and heavy smoking, in addition to ECOG score, LDH, and NLR, with a cutoff value of 4 for NLR. Further details are shown in Supplementary Tables 1 and 2. The normal range of LDH was 109–245 units/L. The unit of LDH in the original EPSILoN score was mg/ml and the cutoff value was 400. In this paper, considering the clinical practice in China, the unit was changed to U/L and the cutoff value was 1.5*ULN, and the consent of the original authors has been obtained. Heavy smoking was defined as smoking more than or equal to 43 packs a year. LIPI and EPSILoN scores categorized patients into three prognostic groups, being good (best), intermediate, and poor, while mLIPI scores identified four prognostic groups, being good, intermediate, poor, and very poor.

**TABLE 1:** Patient characteristics (*n* = 429)

Feature	*n*	Percentage (%)
Sex		
Male	314	73.2
Female	115	26.8
Age (years)		
<65	288	67.1
≥65	141	32.9
Histology		
Squamous carcinoma	163	38.0
Adenocarcinoma	266	62.0
Clinical stage		
IIIb–c	78	18.1
IV	351	81.8
ECOG‐PS		
0–1	407	94.9
2–3	22	5.1
Smoking status		
Never	224	52.2
Former/current	205	47.8
Line of immunotherapy		
First	74	17.2
Second	167	39.0
≥Third	188	43.8
Actionable mutation		
(−)/undetected	337	78.6
EGFR	53	12.4
ALK	4	0.9
KRAS	24	5.6
TP53	24	5.6
PD‐L1 expression		
(−)/undetected	371	86.5
≥1%, <50%	23	5.4
≥50%	35	8.1
Number of metastatic sites		
<3	354	82.5
≥3	75	17.5
Liver metastases		
Yes	62	14.5
No	367	85.5
Brain metastases		
Yes	102	23.8
No	327	76.2
Bone metastases		
Yes	124	28.9
No	305	71.1
LDH (U/L)		
≤ULN	281	65.5
>ULN	148	34.5
≤1.5^*^ULN	380	88.6
>1.5^*^ULN	49	11.4
NLR		
<3	196	45.7
≥3	233	54.3
NLR		
<4	301	70.2
≥4	128	29.8
dNLR		
≤3	346	80.7
>3	83	19.3
PLR		
<160	167	38.9
≥160	262	61.1
Lines of treatment		
1	74	17.3
2	167	38.9
≥3	188	43.8
Type of immunotherapy		
Nivolumab	42	9.7
Pembrolizumab	50	11.6
Toripalimab	60	14.0
Sintilimab	227	52.9
Camrelizumab	38	8.9
Tislelizumab	12	2.8
Best overall response		
CR	0	0
PR	99	23.1
SD +PD	330	76.9
Overall survival status		
Alive	293	68.3
Deceased	136	31.7

Abbreviations: dNLR, derived neutrophil‐to‐lymphocyte ratio; ECOG‐PS, Eastern Cooperative Oncology Group performance status; LDH, lactate dehydrogenase; NLR, neutrophil‐to‐lymphocyte ratio; PLR, platelet‐to‐lymphocyte ratio; CR, complete response; PR, partial response; SD, stable disease; PD, partial disease.

**TABLE 2:** Univariate analysis of PFS

Covariate	Category	Univariate analysis	
		HR (95% CI)	*p* value
Sex	Female	1.109 (0.861–1.429)	0.427
Age	≥65	0.855 (0.666–1.096)	0.217
Histology	Adenocarcinoma	1.234 (0.971–1.569)	0.085
Clinical stage	IV	1.605 (1.161–2.220)	0.004^*^
ECOG PS	2–3	3.109 (1.966–4.917)	<0.001^*^
Smoking status	Former/current	0.732 (0.581–0.923)	0.008^*^
Number of metastatic sites	≥3	1.852 (1.389–2.469)	<0.001^*^
Line of immunotherapy	≥3	1.401 (1.113–1.764)	0.004^*^
Liver metastases	Yes	1.958 (1.448–2.646)	<0.001^*^
Brain metastases	Yes	1.303 (0.997–1.703)	0.053
Bone metastases	Yes	1.451 (1.133–1.859)	0.003^*^
NLR	≥3	2.736 (2.158–3.470)	<0.001^*^
	≥4	2.890 (2.267–3.683)	<0.001^*^
dNLR	>3	2.504 (1.906–3.290)	<0.001^*^
LDH (U/L)	>ULN	1.873 (1.481–2.370)	<0.001^*^
	≥1.5^*^ULN	3.398 (2.477–4.660)	<0.001^*^
PLR	≥160	1.552 (1.219–1.977)	<0.001^*^

Abbreviations: dNLR, derived neutrophil‐to‐lymphocyte ratio; ECOG PS, Eastern Cooperative Oncology Group performance status; LDH, lactate dehydrogenase; NLR, neutrophil‐to‐lymphocyte ratio; PFS, progression‐free survival; PLR, platelet‐to‐lymphocyte ratio.

^*^ means *p* < 0.05.

### 2.3 Statistical analysis

SPSS 23.0 software (IBM SPSS, Armonk, NY) and R software (version 3.6.0) were used for statistical analyses. Differences between the groups were compared using Kruskal–Wallis H tests. The discriminative ability of the predictive models was evaluated by AUC in the ROC analysis. The predictive accuracy was evaluated by calculating the concordance index (C‐index) of each score. Hosmer–Lemeshow tests (H–L tests) were conducted to determine the fit of the prediction models, and Spearman's correlation coefficients were calculated to assess the association between predictive model scores and survival time. PFS and OS curves were generated using the Kaplan–Meier method and differences were assessed using log‐rank tests. Univariate and multivariate analyses were performed using Cox proportional hazards regression models. Two‐tailed *p* values of less than 0.05 were considered to be statistically significant.

## 3 RESULTS

### 3.1 Patient characteristics

The baseline clinical characteristics of the 429 patients are shown in Table 1. The median age was 61 years with a range of 30–79 years, and 32.9% of patients were aged 65 or older. Most of the patients were men (*n* = 314; 73.2%), and approximately half were heavy smokers (*n* = 224; 52.2%). Adenocarcinoma was the predominant histologic subtype (*n* = 266, 62.0%), and more than half of the patients had a good performance status, with ECOG PS values of 0 and 1 reported by 407 of the patients, being 94.9%. At the time of treatment, liver, brain, and bone metastases had occurred in 14.5%, 23.8%, and 28.9% of patients, respectively. Of the 429 patients, 74 (17.3%) received immunotherapy as first‐line treatment. At the time of statistical analysis, 293 patients (68.3%) had disease progression, and 138 patients (38.2%) were deceased. Following a median follow‐up period of 9.9 months, the median OS and PFS were 14.2 months and 5.6 months, respectively. The ORR was 23.1% [*n* = 99, 95% confidence interval (CI): 19.1–27.1].

### 3.2 Survival analysis based on LIPI, mLIPI, and EPSILoN scores

The variables included in the univariate analysis for PFS and OS are shown in Tables 2 and 3. In multivariate model, ECOG PS (HR 2.521, 95% CI: 1.6–3.1; *p* = 0.001), heavy smoking (HR 0.790, 95% CI: 0.6–1.0; *p* = 0.048), liver metastases (HR 1.567, 95% CI: 1.1–2.1; *p* = 0.009), dNLR>3 (HR 1.984, 95% CI: 1.5–2.7; *p* < 0.001), and LDH>ULN (HR 1.666, 95% CI: 1.3–2.1; *p* < 0.001) were shown to be independent prognostic biomarkers for PFS of LIPI score. ECOG PS (HR 2.386, 95% CI: 1.5–3.9; *p* < 0.001), heavy smoking (HR 0.705, 95% CI: 0.6–0.9; *p* = 0.048), liver metastases (HR 1.838, 95% CI: 1.3–2.6; *p* < 0.001), NLR≥3 (HR 3.340, 95% CI: 2.5–4.4; *p* < 0.001), and LDH>1.5*ULN (HR 2.661, 95% CI: 1.8–3.7; *p* < 0.001) were shown to be independent prognostic biomarkers for PFS of mLIPI score. ECOG PS (HR 2.199, 95% CI: 1.4–3.6; *p* = 0.001), heavy smoking (HR 0.759, 95% CI: 0.6–1.0; *p* = 0.021), liver metastases (HR 1.677, 95% CI: 1.2–2.3; *p* = 0.002), NLR≥4 (HR 2.455, 95% CI: 1.9–3.2; *p* < 0.001), and LDH>1.5*ULN (HR 2.715, 95% CI: 1.9–3.9; *p* < 0.001) were shown to be independent prognostic biomarkers for PFS of EPSILoN score (Tables 4, 5, 6). The variables included in the multivariate analysis for the OS of these three scores are shown in Tables 7, 8, 9.

**TABLE 3:** Univariate analysis of OS

Covariate	Category	Univariate analysis	
		HR (95% CI)	*p* value
Sex	Female	1.024 (0.702–1.495)	0.900
Age	≥65	1.198 (0.844–1.700)	0.313
Histology	Adenocarcinoma	1.368 (0.955–1.960)	0.088
Clinical stage	IV	1.926 (1.143–3.246)	0.014*
ECOG PS	2–3	7.138 (4.311–11.818)	<0.001*
Smoking status	Former/current	1.035 (0.739–1.450)	0.839
Number of metastatic sites	≥3	1.359 (1.187–1.556)	<0.001*
Line of immunotherapy	≥3	1.668 (1.193–2.330)	0.003*
Liver metastases	Yes	1.893 (1.255–2.855)	0.002*
Brain metastases	Yes	1.589 (1.087–2.323)	0.017*
Bone metastases	Yes	2.010 (1.425–2.836)	<0.001*
NLR	≥3	3.878 (2.623–5.733)	<0.00^1*^
	≥4	2.729 (1.936–3.847)	<0.001*
dNLR	>3	2.522 (1.740–3.656)	<0.001*
LDH	>ULN	2.640 (1.887–3.693)	<0.001*
	>1.5*ULN	4.554 (3.066–6.764)	<0.001*
PLR	≥160	1.382 (0.971–1.966)	0.072

Abbreviations: dNLR, derived neutrophil‐to‐lymphocyte ratio; ECOG PS, Eastern Cooperative Oncology Group performance status; LDH, lactate dehydrogenase; NLR, neutrophil‐to‐lymphocyte ratio; OS, overall survival; PLR, platelet‐to‐lymphocyte ratio.

* *p* < 0.05.

**TABLE 4:** Multivariate analysis of PFS for LIPI

Covariate	Category	Multivariate analysis	
		HR (95% CI)	*p* value
Clinical stage	IV	1.232 (0.872–1.741)	0.237
ECOG PS	2–3	2.521 (1.554–3.090)	0.001^*^
Smoking status	Former/current	0.790 (0.625–0.998)	0.048^*^
Number of metastatic sites	≥3	1.393(0.991–1.957)	0.056
Line of immunotherapy	≥3	1.194 (0.940–1.515)	0.146
Liver metastases	Yes	1.567 (1.117–2.199)	0.009^*^
Bone metastases	Yes	0.935 (0.701–1.247)	0.646
dNLR	>3	1.984 (1.479–2.659)	<0.001^*^
LDH	>ULN	1.666 (1.298–2.140)	<0.001^*^
PLR	≥160	1.284 (0.992–1.661)	0.058

Abbreviations: dNLR, derived neutrophil‐to‐lymphocyte ratio; ECOG PS, Eastern Cooperative Oncology Group performance status; LDH, lactate dehydrogenase; NLR, neutrophil‐to‐lymphocyte ratio; PFS, progression‐free survival; PLR, platelet‐to‐lymphocyte ratio.

* means *p* < 0.05.

**TABLE 5:** Multivariate analysis of PFS for mLIPI

Covariate	Category	Multivariate analysis	
		HR (95% CI)	*p* value
Clinical stage	IV	1.226 (0.864–1.740)	0.253
ECOG PS	2–3	2.386 (1.476–3.859)	<0.001^*^
Smoking status	Former/current	0.705 (0.557–0.892)	0.004^*^
Number of metastatic sites	≥3	1.280(0.905–1.810)	0.163
Line of immunotherapy	≥3	1.175 (0.924–1.495)	0.187
Liver metastases	Yes	1.838 (1.313–2.573)	<0.001^*^
Bone metastases	Yes	0.958 (0.714–1.285)	0.776
NLR	≥3	3.340 (2.548–4.377)	<0.001^*^
LDH	>1.5^*^ULN	2.611 (1.835–3.714)	<0.001^*^
PLR	≥160	0.935 (0.717–1.219)	0.620

Abbreviations: dNLR, derived neutrophil‐to‐lymphocyte ratio; ECOG PS, Eastern Cooperative Oncology Group performance status; LDH, lactate dehydrogenase; NLR, neutrophil‐to‐lymphocyte ratio; PFS, progression‐free survival; PLR, platelet‐to‐lymphocyte ratio.

* means *p* < 0.05.

**TABLE 6:** Multivariate analysis of PFS for EPSILoN

Covariate	Category	Multivariate analysis	
		HR (95% CI)	*p* value
Clinical stage	IV	1.255 (0.886–1.777)	0.201
ECOG PS	2–3	2.199 (1.356–3.565)	0.001^*^
Smoking status	Former/current	0.759 (0.601–0.959)	0.021^*^
Number of metastatic sites	≥3	1.263(0.894–1.785)	0.186
Line of immunotherapy	≥3	1.245 (0.979–1.584)	0.074
Liver metastases	Yes	1.677 (1.200–2.343)	0.002^*^
Bone metastases	Yes	0.910 (0.678–1.222)	0.531
NLR	≥4	2.455 (1.875–3.214)	< 0.001^*^
LDH	>1.5^*^ULN	2.715 (1.904–3.872)	<0.001^*^
PLR	≥160	1.074 (0.820–1.408)	0.604

Abbreviations: dNLR, derived neutrophil‐to‐lymphocyte ratio; ECOG PS, Eastern Cooperative Oncology Group performance status; LDH, lactate dehydrogenase; NLR, neutrophil‐to‐lymphocyte ratio; PFS, progression‐free survival; PLR, platelet‐to‐lymphocyte ratio.

* means *p* < 0.05.

**TABLE 7:** Multivariate analysis of OS for LIPI

Covariate	Category	Multivariate analysis	
		HR (95% CI)	*p* value
Clinical stage	IV	1.247 (0.711–2.186)	0.441
ECOG PS	2–3	4.875 (2.800–8.485)	<0.001*
Number of metastatic sites	≥3	1.772(1.089–2.883)	0.021*
Line of immunotherapy	≥3	1.475 (1.028–2.065)	0.034*
Liver metastases	Yes	1.198 (0.747–1.923)	0.453
Brain metastases	Yes	1.063(0.704–1.605)	0.771
Bone metastases	Yes	1.057 (0.705–1.587)	0.787
dNLR	>3	1.860 (1.260–2.747)	0.002*
LDH	>ULN	2.025(1.416–2.896)	<0.001*

Abbreviations: dNLR, derived neutrophil‐to‐lymphocyte ratio; ECOG PS, Eastern Cooperative Oncology Group performance status; LDH, lactate dehydrogenase; NLR, neutrophil‐to‐lymphocyte ratio; OS, overall survival; PLR, platelet‐to‐lymphocyte ratio.

* *p* < 0.05.

**TABLE 8:** Multivariate analysis of OS for mLIPI

Covariate	Category	Multivariate analysis	
		HR (95% CI)	*p* value
Clinical stage	IV	1.228 (0.694–2.173)	0.428
ECOG PS	2–3	4.753 (2.749–8.220)	<0.001*
Number of metastatic sites	≥3	1.442(0.864–2.405)	0.161
Line of immunotherapy	≥3	1.474(1.041–2.088)	0.029*
Liver metastases	Yes	1.435 (0.892–2.308)	0.137
Brain metastases	Yes	1.218(0.804–1.844)	0.352
Bone metastases	Yes	1.152(0.761–1.745)	0.503
NLR	≥3	3.512 (2.353–5.241)	<0.001*
LDH	>1.5*ULN	2.995(1.923–4.663)	<0.001*

Abbreviations: dNLR, derived neutrophil‐to‐lymphocyte ratio; ECOG PS, Eastern Cooperative Oncology Group performance status; LDH, lactate dehydrogenase; NLR, neutrophil‐to‐lymphocyte ratio; OS, overall survival; PLR, platelet‐to‐lymphocyte ratio.

* *p* < 0.05.

**TABLE 9:** Multivariate analysis of OS for EPSILoN

Covariate	Category	Multivariate analysis	
		HR (95% CI)	*p* value
Clinical stage	IV	1.284 (0.728–2.262)	0.388
ECOG PS	2–3	4.909 (2.838–8.493)	<0.001*
Number of metastatic sites	≥3	1.444(0.872–2.391)	0.153
Line of immunotherapy	≥3	1.485 (1.049–2.102)	0.026*
Liver metastases	Yes	1.355 (0.842–2.180)	0.210
Brain metastases	Yes	1.238(0.822–1.862)	0.307
Bone metastases	Yes	1.097 (0.727–1.655)	0.659
NLR	≥4	2.091 (1.457–3.001)	<0.001*
LDH	>1.5*ULN	3.020(1.939–4.703)	<0.001*

Abbreviations: dNLR, derived neutrophil‐to‐lymphocyte ratio; ECOG PS, Eastern Cooperative Oncology Group performance status; LDH, lactate dehydrogenase; NLR, neutrophil‐to‐lymphocyte ratio; OS, overall survival; PLR, platelet‐to‐lymphocyte ratio.

* *p* < 0.05.

**FIGURE 1:**
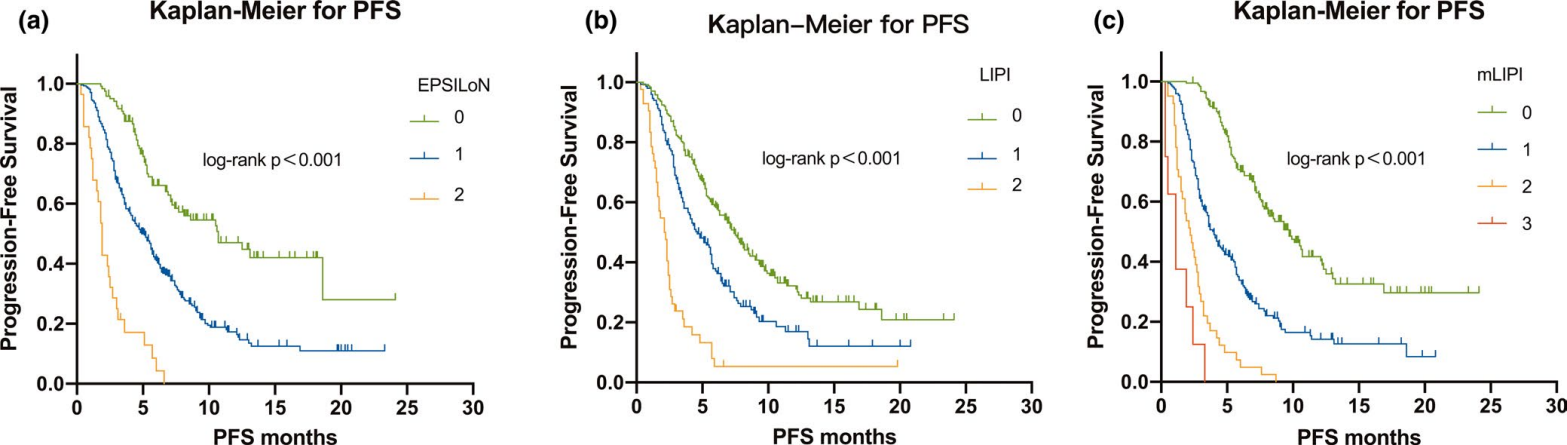
Kaplan–Meier analysis of PFS. (A) PFS rate for LIPI; (B) PFS rate for mLIPI; and (C) PFS rate for EPSILoN. PFS, progression‐free survival; LIPI, lung immune prognostic index; mLIPI, modified lung immune predictive index; EPSILoN, Eastern Cooperative Oncology Group performance status, smoking, liver metastases, lactate dehydrogenase, neutrophil‐to‐lymphocyte ratio

**FIGURE 2:**
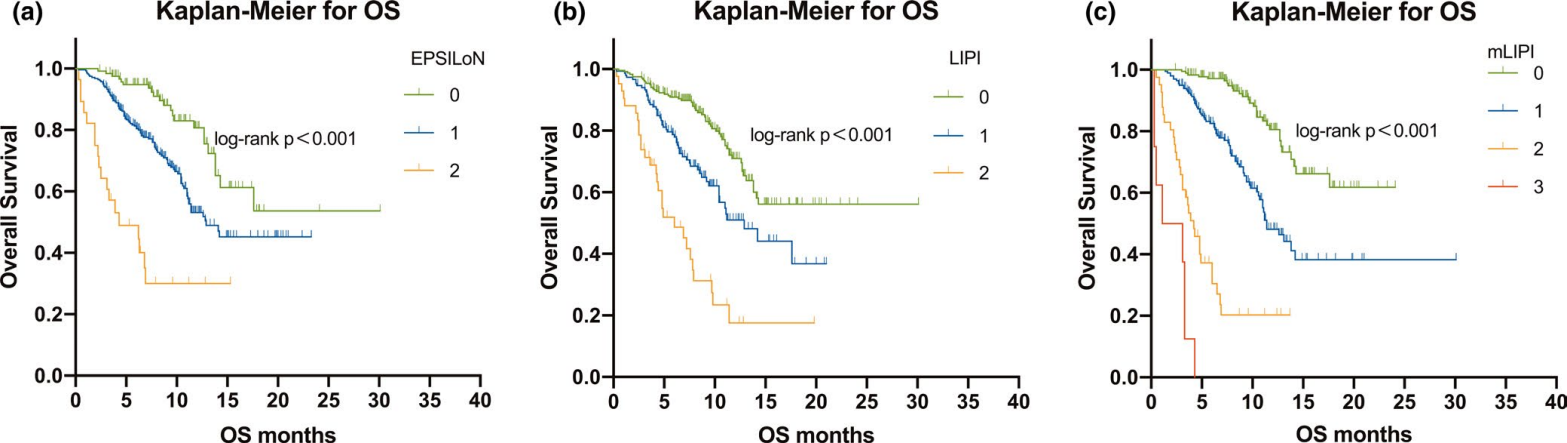
Kaplan–Meier analysis of OS. (A) OS rate for LIPI; (B) OS rate for mLIPI; (C) and OS rate for EPSILoN. N/A, not available. OS, overall survival; LIPI, lung immune prognostic index; mLIPI, modified lung immune predictive index; EPSILoN, Eastern Cooperative Oncology Group performance status, smoking, liver metastases, lactate dehydrogenase, neutrophil‐to‐lymphocyte ratio

**FIGURE 3:**
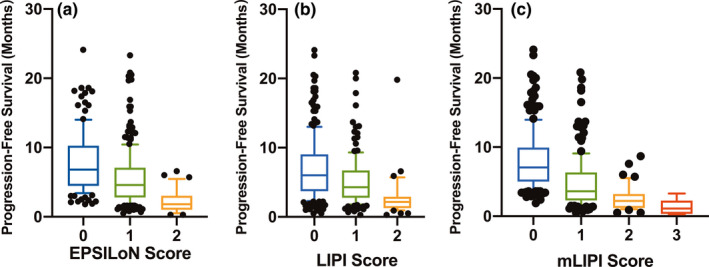
Associations between the three predictive tools and progression‐free survival (PFS)

We then analyzed the patient outcomes based on the three predictive models. Patients with higher scores on these models, whether on LIPI, mLIPI, or EPSILoN, demonstrated more risk factors and significantly shorter PFS and OS (*p* < 0.001 for all models) (Figures 1, 2, 3).

### 3.3 Predictive ability of LIPI, mLIPI, and EPSILoN scores for PFS and ORR

**FIGURE 4:**
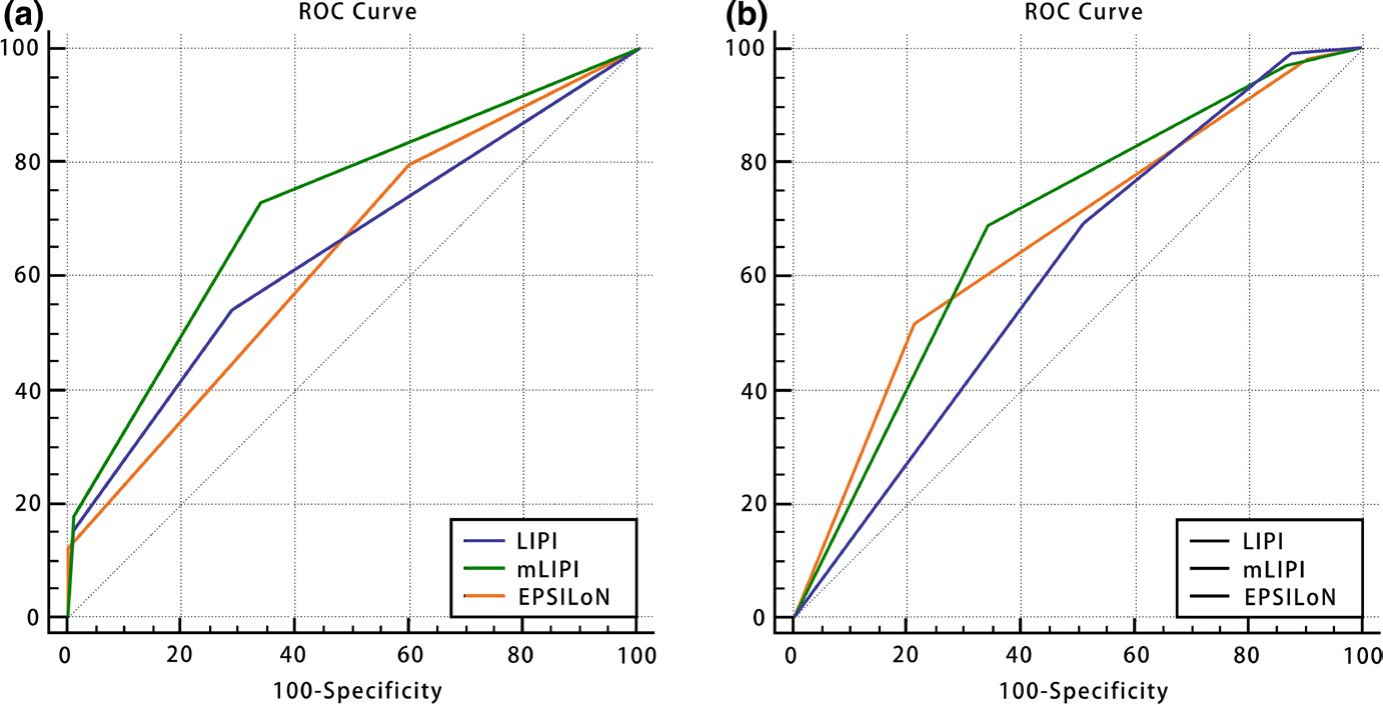
ROC curves of (A) PFS predictions by LIPI, mLIPI, and EPSILoN scores at 6 months and (B) ORR. ROC, receiver operating characteristic curve; PFS, progression‐free survival; LIPI, lung immune prognostic index; mLIPI, modified lung immune predictive index; EPSILoN, Eastern Cooperative Oncology Group performance status, smoking, liver metastases, lactate dehydrogenase, neutrophil‐to‐lymphocyte ratio; ORR, objective response rate

The AUC values of LIPI, mLIPI, and EPSILoN scores for predicting PFS at 6 months were 0.642 (95% CI:0.590–0.694), 0.720 (95% CI: 0.675–0.762), and 0.633 (95% CI: 0.585–0.679), with sensitivities of 53.6%, 72.6%, and 79.5% and specificities of 71.1%, 66.3%, and 40.4%, respectively (*p* < 0.001 for all models). The AUC value of mLIPI scores was significantly higher than that of LIPI and EPSILoN scores (*p* < 0.05). The C‐indexes of LIPI, mLIPI, and EPSILoN scores for PFS were 0.627 (95% CI 0.611–6.643), 0.677 (95% CI 0.652–0.682), and 0.631 (95% CI 0.617–0.645), respectively. The mLIPI model demonstrated the best predictive power for PFS. The Hosmer–Lemeshow chi‐squared values of LIPI, mLIPI, and EPSILoN scores were 6.142, 7.982, and 4.333, respectively (*p* > 0.05 for all models). The Spearman's correlation coefficients calculated between observed and expected clinical results were −0.355, −0.560, and −0.369, respectively (*p* < 0.001 for all models) (Tables 10 and 11) (Figure 4). The AUC values of LIPI, mLIPI, and EPSILoN scores for predicting the ORR were 0.606 (95% CI: 0.546–0.665), 0.683 (95% CI: 0.637–0.727), and 0.666 (95% CI: 0.637–0.711), with sensitivities of 69.7%, 68.7%, and 51.5% and specificities of 48.2%, 65.5%, and 78.8%, respectively (*p* < 0.001 for all models) (Tables 12 and 13) (Figure 4). The mLIPI model demonstrated the best predictive power for ORR.

**TABLE 10:** PFS prediction at 6 months by LIPI, mLIPI, and EPSILoN scores

Predictive models	AUC (95% CI)	C‐index(95% CI)	Spearman rank correlation (*p* value)	H–L test (*p* value)
LIPI	0.642 (0.590–0.694)	0.627 (0.611–6.643)	−0.355 (<0.001)	6.142 (0.631)
mLIPI	0.720 (0.675–0.762)	0.677 (0.652–0.682)	−0.560 (<0.001)	7.982 (0.435)
EPSILoN	0.633 (0.585–0.679)	0.631 (0.617–0.645)	−0.369 (<0.001)	4.333 (0.826)

Abbreviations: EPSILoN, Eastern Cooperative Oncology Group performance status, smoking, liver metastases, lactate dehydrogenase, neutrophil‐to‐lymphocyte ratio; LIPI, lung immune prognostic index; mLIPI, modified lung immune predictive index; PFS, progression‐free survival.

**TABLE 11:** Comparison of ROC curves of PFS prediction at 6 months by LIPI, mLIPI, and EPSILoN scores

Predictive models	*z* value	*p* value
LIPI vs. mLIPI	2.928	0.0034^*^
mLIPI vs. EPSILoN	3.393	0.0007^*^
EPSILoN vs. LIPI	0.470	0.6385

Abbreviations: EPSILoN, Eastern Cooperative Oncology Group performance status, smoking, liver metastases, lactate dehydrogenase, neutrophil‐to‐lymphocyte ratio; LIPI, lung immune prognostic index; mLIPI, modified lung immune predictive index; ROC, receiver operating characteristic curve; PFS, progression‐free survival.

*means *p* < 0.05.

**TABLE 12:** ORR predictions at 6 months by LIPI, mLIPI, and EPSILoN scores

Predictive models	AUC (95% CI)
LIPI	0.606 (0.546–0.665)
mLIPI	0.683 (0.637–0.727)
EPSILoN	0.666 (0.620–0.711)

Abbreviations: AUC, area under the curve; EPSILoN, Eastern Cooperative Oncology Group performance status, smoking, liver metastases, lactate dehydrogenase, neutrophil‐to‐lymphocyte ratio; LIPI, lung immune prognostic index; mLIPI, modified lung immune predictive index; ORR, objective response rate.

**TABLE 13:** Comparison of ROC curves of ORR predictions at 6 months by LIPI, mLIPI, and EPSILoN scores

Predictive models	*z* value	*p* value
LIPI vs. mLIPI	2.510	0.0121^*^
mLIPI vs. EPSILoN	0.517	0.6054
EPSILoN vs. LIPI	1.852	0.0641

Abbreviations: EPSILoN, Eastern Cooperative Oncology Group performance status, smoking, liver metastases, lactate dehydrogenase, neutrophil‐to‐lymphocyte ratio; LIPI, lung immune prognostic index; mLIPI, modified lung immune predictive index; ORR, objective response rate; ROC, receiver operating characteristic curve.

* means *p* < 0.05.

## 4 DISCUSSION

The advent of immunotherapy has significantly increased the long‐term survival for patients with aNSCLC. However, oncologists have not yet reached a consensus on characteristics that define the patient population which most benefits from immunotherapy. Although PD‐L1 expression is currently the most widely accepted biomarker for predicting the efficacy of immunotherapy, its expression status can be modified by prior treatments^22^ and some studies have shown PD‐L1 expression in diagnostic biopsy tissues and surgical specimens to be highly inconsistent,^23^ the use of the expression of PD‐L1 to predict if patients will respond to immunotherapy is unreliable.

Since Rudolf Virchow first detected leukocytes in tumor tissues in the early 19^th^ century, the potential relationship between tumors and inflammation has attracted widespread attention.^24^ Epidemiological surveys show that cancers caused by chronic infection and inflammation account for 25% of all cancers, including lung cancer.^25^ While acute inflammation attacks tumors, chronic inflammation suppresses immune defense against tumors,^26^ and NLR has been shown to reflect the degree of chronic inflammation in patients.^27^ Lymphocytes play a key role in recognizing and killing tumors, and also assist ICIs in performing their antitumor functions. Complex bidirectional interactions have been observed between neutrophils and tumor cells: neutrophils secrete chemokines and cytokines to produce an immunosuppressive environment that favors tumor proliferation, while tumor cells release granulocyte‐colony stimulating factor, which promotes the aggregation of neutrophils. Later studies have reported that neutrophils may provide a significant source of matrix metallopeptidase 9 (MMP‐9). This drives angiogenesis in malignant tumors, contributing to tumor invasion and metastasis.^28^ Arginine has been previously reported to be an essential amino acid for a variety of cellular processes, including T‐cell proliferation. Neutrophils can aid tumor invasion by expressing arginase, which degrades arginine and affects the proliferation of T cells.^29^ NLR could, therefore, be used as an indicator to reflect a patient's inflammation and immune status. While dynamic changes in NLR have been shown to predict the prognosis of immunotherapy‐treated patients in many studies, a cutoff value has not yet been agreed between these studies.^14^, ^30^, ^31^, ^32^, ^33^ In our study, NLR ≥3 was significantly associated with poor PFS and OS. However, some studies indicated that not all neutrophils and tumor cells had a mutually reinforcing relationship. Due to the increased expression of tumor necrosis factor‐alpha (TNF‐α) and intercellular adhesion molecule 1 (ICAM‐1), N1 (anti‐tumor) phenotype neutrophils are cytotoxic toward tumor cells.^34^ The next challenge is, therefore, to identify specific neutrophil subpopulations that can be used to accurately predict the efficacy of immunotherapy.

LDH is produced by fast‐growing tumor cells and can reflect the tumor burden and inflammatory state of patients with solid tumors. Multiple studies have demonstrated that high LDH levels are significantly correlated with poor prognosis in patients treated with ICIs. This may be due to the accumulation of serum tumor‐derived lactic acid, which contributes to the inability of CD8 T cells to export lactate, leading to metabolic disorders. Derangement of T‐cell metabolism might be another mechanism of tumor resistance to ICIs.^35^, ^36^

Previous randomized clinical trials of immunotherapy in NSCLC have generally only included patients with ECOG PS values of 0 and 1. In clinical practice, however, 18% of lung cancer patients are PS values of 2. It is uncertain whether these patients with poor clinical performance can benefit from immunotherapy. Patients with poor clinical performance usually have frailer immune systems and lower survival expectations. Past research has shown that poor ECOG PS is associated with poor prognosis in patients with NSCLC.^19^, ^20^, ^21^ In our study, PS ≥2 was found to be an independent risk factor for poor prognosis. A recent prospective trial, however, included 60 NSCLC patients with a rigorous ascription of PS 2 receiving pembrolizumab. This study showed that pembrolizumab was as effective in treating PS 2 patients as it was treating PS 0–1 patients.^37^ Further prospective studies are required to investigate whether ECOG PS can be used as a prognostic biomarker to predict the efficacy of immunotherapy.

Liver metastases are associated with poor patient outcomes and occurs in approximately one fifth of NSCLC cases. Whether patients affected by NSCLC with liver metastasis can benefit from immunotherapy alone remains unknown. Several retrospective studies have reported that NSCLC patients with liver metastases receiving ICI monotherapy have a poor prognosis.^38^, ^39^ Additionally, the Phase I CA209‐003 trial demonstrated that liver metastases were independently associated with a reduction in 5‐year survival rate.^40^ However, the CheckMate‐017 and CheckMate‐057 trials showed that nivolumab treatment alone had survival benefits for NSCLC patients with liver metastases. One meta‐analysis included 4485 patients, yielded summary statistics indicating that lung cancer patients with liver metastases could also benefit from immunotherapy alone, but they would have relatively less benefit than those without liver metastases. KEYNOTE‐189 showed that pembrolizumab combined with chemotherapy could improve the PFS and OS of lung cancer with liver metastases. The subgroup analysis of IMpower150 demonstrated that who received the addition of immunotherapy to bevacizumab plus chemotherapy achieved more clinical benefits than those without liver metastases.^41^ As our study indicated that, according to EPSILoN scores, liver metastases are a risk factor for patients not responding to immunotherapy, this requires further exploration.

Heavy smoking was shown in our study to be a positive prognostic biomarker for immunotherapy response. Several clinical trials have reported that smokers seem to benefit more from immunotherapy than non‐smokers.^42^, ^43^, ^44^ Smoking‐related lung cancer is associated with a higher mutational burden and PD‐L1 expression level, which is consistent with an increased efficacy of immunotherapy.^45^, ^46^ Animal model studies have shown that tobacco enriches CD4‐, CD8‐, and PD‐1‐positive lymphocytes in the lungs of mice, which possibly explain the increased response of smokers to immunotherapy.^47^ However, Pirker states that smoking history should not be considered a predictive marker for immunotherapy. First, a subgroup analysis of phase III clinical trials found that associations between smoking history and the benefit of ICI treatment were inconsistent. Second, more than 80% of lung cancers are smoking‐related in the Western population, which limits the use of smoking history as a biomarker.^48^ However, the usefulness of smoking history in predicting the response of Chinese NSCLC patients to immunotherapy requires further verification. Even if smoking is associated with better efficacy of immunotherapy in NSCLC patients, relaxing global tobacco control is not recommended because the benefits of no smoking would be more valuable in reducing global lung cancer mortality than any cancer treatment.

The clinical parameters included in the above three prediction models were all routinely examined and easily available in clinical practice. External validation of the three scores also confirmed that higher scores were significantly associated with shorter PFS and OS, and mLIPI had the highest predictive power for PFS and ORR. The advantages of these three predictive models were obvious, as all the clinical parameters included are simple, non‐invasive, and easily accessible. For the EPSILoN score, it included the largest number of indicators and each of them had an impact on the prognosis of immunotherapy, but the predictive accuracy was not the highest, probably because the score treated the predictive power of each independent risk factor affecting prognosis as equal, which may lead to overestimation or underestimation of individual predictors. Moreover, based on the previous analysis, although heavy smoking was found to be a protective factor for the prognosis of immunotherapy in this study, its inclusion in the scoring model needs to be cautious, considering its actual clinical significance and the controversial predictive role of existing clinical studies. The inclusion of liver metastases as an adverse risk factor for immunotherapy prognosis was also controversial, and there were large randomized controlled clinical studies demonstrating that NSCLC patients with liver metastases may also benefit from immunotherapy. In conclusion, the mLIPI score had the best predictive efficacy from the statistical analysis, and from the clinical significance, the parameters included were relatively comprehensive and less controversial, and the calculation method was simple and convenient, which could help doctors make preliminary judgments quickly in clinical application.

Our study also has several limitations. First of all, this is a retrospective, single‐center research with a relatively small sample size. Therefore, a multi‐center and larger sample size is required for further verification. Furthermore, a control group without immunotherapy is needed to further verify the predictive value of these three scoring methods on PFS and ORR. Finally, most patients received immunotherapy as second‐line or beyond. Although the number of lines of immunotherapy was not related to the clinical outcomes, the previous treatment may affect the baseline parameters.^49^, ^50^, ^51^, ^52^

## 5 CONCLUSIONS

By externally validating LIPI, mLIPI, and EPSILoN scores, we found that all three of these predictive models could identify different prognostic subsets of patients treated with ICIs to statistically significant degrees. We also found that mLIPI had the highest accuracy of the three models for Chinese aNSCLC patients in predicting PFS, ORR, and the clinical significance of included indicators. This predictive tool might, therefore, be useful in guiding clinical immunotherapy decision‐making.

## CONFLICT OF INTEREST

The authors have no conflict of interest to declare.

## Supporting information

Table S1‐S2

## Data Availability

The raw data supporting the conclusions of this article will be made available by Linlin Wang (wanglinlinatjn@163.com) for a period of 5 years after the publication date.

## References

[1] Vansteenkiste J, Wauters E, Reymen B, et al. Current status of immune checkpoint inhibition in early‐stage NSCLC. Ann Oncol. 2019;30:1244‐1253. 31143921 10.1093/annonc/mdz175

[2] Brahmer J, Reckamp KL, Baas P, et al. Nivolumab versus Docetaxel in Advanced Squamous‐Cell Non‐Small‐Cell Lung Cancer. N Engl J Med. 2015;373:123‐135. 26028407 10.1056/NEJMoa1504627 PMC4681400

[3] Borghaei H, Paz‐Ares L, Horn L, et al. Nivolumab versus docetaxel in advanced nonsquamous non‐small‐cell lung cancer. N Engl J Med. 2015;373:1627‐1639. 26412456 10.1056/NEJMoa1507643 PMC5705936

[4] Horn L, Spigel DR, Vokes EE, et al. nivolumab versus docetaxel in previously treated patients with advanced non‐small‐cell lung cancer: two‐year outcomes from two randomized, open‐label, phase III trials (CheckMate 017 and CheckMate 057). J Clin Oncol. 2017;35:3924‐3933. 29023213 10.1200/JCO.2017.74.3062 PMC6075826

[5] Reck M, Rodríguez‐Abreu D, Robinson AG, et al. Pembrolizumab versus chemotherapy for PD‐L1–positive non–small‐cell lung cancer. N engl J med. 2016;375:1823‐1833. 27718847 10.1056/NEJMoa1606774

[6] Herbst RS, Baas P, Kim DW, et al. Pembrolizumab versus docetaxel for previously treated, PD‐L1‐positive, advanced non‐small‐cell lung cancer (KEYNOTE‐010): a randomised controlled trial. Lancet. 2016;387:1540‐1550. 26712084 10.1016/S0140-6736(15)01281-7

[7] Rittmeyer A, Barlesi F, Waterkamp D, et al. Atezolizumab versus docetaxel in patients with previously treated non‐small‐cell lung cancer (OAK): a phase 3, open‐label, multicentre randomised controlled trial. Lancet. 2017;389:255‐265. 27979383 10.1016/S0140-6736(16)32517-X PMC6886121

[8] Vokes EE, Ready N, Felip E, et al. Nivolumab versus docetaxel in previously treated advanced non‐small‐cell lung cancer (CheckMate 017 and CheckMate 057): 3‐year update and outcomes in patients with liver metastases. Ann Oncol. 2018;29:959‐965. 29408986 10.1093/annonc/mdy041

[9] Reck M, Rodriguez‐Abreu D, Robinson AG, et al. Updated analysis of KEYNOTE‐024: pembrolizumab versus platinum‐based chemotherapy for advanced non‐small‐cell lung cancer with PD‐L1 tumor proportion score of 50% or greater. J Clin Oncol. 2019;37:537‐546. 30620668 10.1200/JCO.18.00149

[10] Chae YK, Pan A, Davis AA, et al. Biomarkers for PD‐1/PD‐L1 blockade therapy in non‐small‐cell lung cancer: is PD‐L1 expression a good marker for patient selection? Clin Lung Cancer. 2016;17:350‐361. 27137346 10.1016/j.cllc.2016.03.011

[11] Gibney GT, Weiner LM, Atkins MB. Predictive biomarkers for checkpoint inhibitor‐based immunotherapy. Lancet Oncol. 2016;17:e542‐e551. 27924752 10.1016/S1470-2045(16)30406-5 PMC5702534

[12] Prelaj A, Tay R, Ferrara R, et al. Predictive biomarkers of response for immune checkpoint inhibitors in non‐small‐cell lung cancer. Eur J Cancer. 2019;106:144‐159. 30528799 10.1016/j.ejca.2018.11.002

[13] Bagley SJ, Kothari S, Aggarwal C, et al. Pretreatment neutrophil‐to‐lymphocyte ratio as a marker of outcomes in nivolumab‐treated patients with advanced non‐small‐cell lung cancer. Lung Cancer. 2017;106:1‐7. 28285682 10.1016/j.lungcan.2017.01.013

[14] Nakaya A, Kurata T, Yoshioka H, et al. Neutrophil‐to‐lymphocyte ratio as an early marker of outcomes in patients with advanced non‐small‐cell lung cancer treated with nivolumab. Int J Clin Oncol. 2018;23:634‐640. 29442281 10.1007/s10147-018-1250-2 PMC6097082

[15] Sacdalan DB, Lucero JA, Sacdalan DL. Prognostic utility of baseline neutrophil‐to‐lymphocyte ratio in patients receiving immune checkpoint inhibitors: a review and meta‐analysis. Onco Targets Ther. 2018;11:955‐965. 29503570 10.2147/OTT.S153290 PMC5827677

[16] Tanizaki J, Haratani K, Hayashi H, et al. Peripheral blood biomarkers associated with clinical outcome in non‐small cell lung cancer patients treated with nivolumab. J Thorac Oncol. 2018;13:97‐105. 29170120 10.1016/j.jtho.2017.10.030

[17] Agulló‐Ortuño MT, Gomez‐Martin O, Ponce S, et al. Blood predictive biomarkers for patients with non‐small‐cell lung cancer associated with clinical response to nivolumab. Clin Lung Cancer. 2020;21:75‐85. 31562055 10.1016/j.cllc.2019.08.006

[18] Mezquita L, Auclin E, Ferrara R, et al. Association of the lung immune prognostic index with immune checkpoint inhibitor outcomes in patients with advanced non‐small cell lung cancer. JAMA Oncol. 2018;4:351‐357. 29327044 10.1001/jamaoncol.2017.4771 PMC5885829

[19] Moor R, O’Byrne K, Roberts K. Modified lung immune predictive index (mLIPI) as a predictive tool of nivolumab outcomes and immune related adverse events in advanced non‐small cell lung cancer (NSCLC) patients. Lung Cancer. 2019;127:S67‐S68.

[20] Prelaj A, Rebuzzi SE, Pizzutilo P, et al. EPSILoN: a prognostic score using clinical and blood biomarkers in advanced non‐small‐cell lung cancer treated with immunotherapy. Clin Lung Cancer. 2020;21:365‐377.e5. 32245624 10.1016/j.cllc.2019.11.017

[21] Prelaj A, Ferrara R, Rebuzzi SE, et al. EPSILoN: a prognostic score for immunotherapy in advanced non‐small‐cell lung cancer: a validation cohort. Cancers (Basel). 2019;11:1954. 10.3390/cancers11121954 PMC6966664 31817541

[22] Rodriguez E, Lilenbaum RC. Small cell lung cancer: past, present, and future. Curr Oncol Rep. 2010;12:327‐334. 20632219 10.1007/s11912-010-0120-5

[23] Ilie M, Long‐Mira E, Bence C, et al. Comparative study of the PD‐L1 status between surgically resected specimens and matched biopsies of NSCLC patients reveal major discordances: a potential issue for anti‐PD‐L1 therapeutic strategies. Ann Oncol. 2016;27:147‐153. 26483045 10.1093/annonc/mdv489

[24] Coussens LM, Werb Z. Inflammation and cancer. Nature. 2002;420:860‐867. 12490959 10.1038/nature01322 PMC2803035

[25] Hussain SP, Harris CC. Inflammation and cancer: an ancient link with novel potentials. Int J Cancer. 2007;121:2373‐2380. 17893866 10.1002/ijc.23173

[26] Elinav E, Nowarski R, Thaiss CA, et al. Inflammation‐induced cancer: crosstalk between tumours, immune cells and microorganisms. Nat Rev Cancer. 2013;13:759‐771. 24154716 10.1038/nrc3611

[27] Russo A, Russano M, Franchina T, et al. Neutrophil‐to‐lymphocyte ratio (NLR), platelet‐to‐lymphocyte ratio (PLR), and outcomes with nivolumab in pretreated non‐small cell lung cancer (NSCLC): a large retrospective multicenter study. Adv Ther. 2020;37:1145‐1155. 32002809 10.1007/s12325-020-01229-w

[28] Deryugina EI, Zajac E, Juncker‐Jensen A, et al. Tissue‐infiltrating neutrophils constitute the major in vivo source of angiogenesis‐inducing MMP‐9 in the tumor microenvironment. Neoplasia. 2014;16:771‐788. 25379015 10.1016/j.neo.2014.08.013 PMC4212255

[29] Rodriguez PC, Quiceno DG, Zabaleta J, et al. Arginase I production in the tumor microenvironment by mature myeloid cells inhibits T‐cell receptor expression and antigen‐specific T‐cell responses. Cancer Res. 2004;64:5839‐5849. 15313928 10.1158/0008-5472.CAN-04-0465

[30] Fukui T, Okuma Y, Nakahara Y, et al. Activity of nivolumab and utility of neutrophil‐to‐lymphocyte ratio as a predictive biomarker for advanced non‐small‐cell lung cancer: a prospective observational study. Clin Lung Cancer. 2019;20:208‐214.e2. 29803573 10.1016/j.cllc.2018.04.021

[31] Ren F, Zhao T, Liu B, et al. Neutrophil‐lymphocyte ratio (NLR) predicted prognosis for advanced non‐small‐cell lung cancer (NSCLC) patients who received immune checkpoint blockade (ICB). Onco Targets Ther. 2019;12:4235‐4244. 31239702 10.2147/OTT.S199176 PMC6554525

[32] Lalani AA, Xie W, Martini DJ, et al. Change in neutrophil‐to‐lymphocyte ratio (NLR) in response to immune checkpoint blockade for metastatic renal cell carcinoma. J Immunother Cancer. 2018;6:5. 29353553 10.1186/s40425-018-0315-0 PMC5776777

[33] Rossi S, Toschi L, Finocchiaro G, et al. Neutrophil and lymphocyte blood count as potential predictive indicators of nivolumab efficacy in metastatic non‐small‐cell lung cancer. Immunotherapy. 2020;12:715‐724. 32522052 10.2217/imt-2019-0154

[34] Andzinski L, Kasnitz N, Stahnke S, et al. Type I IFNs induce anti‐tumor polarization of tumor associated neutrophils in mice and human. Int J Cancer. 2016;138:1982‐1993. 26619320 10.1002/ijc.29945

[35] Brand A, Singer K, Koehl GE, et al. LDHA‐associated lactic acid production blunts tumor immunosurveillance by T and NK cells. Cell Metab. 2016;24:657‐671. 27641098 10.1016/j.cmet.2016.08.011

[36] Diem S, Kasenda B, Spain L, et al. Serum lactate dehydrogenase as an early marker for outcome in patients treated with anti‐PD‐1 therapy in metastatic melanoma. Br J Cancer. 2016;114:256‐261. 26794281 10.1038/bjc.2015.467 PMC4742588

[37] Middleton G, Brock K, Savage J, et al. Pembrolizumab in patients with non‐small‐cell lung cancer of performance status 2 (PePS2): a single arm, phase 2 trial. Lancet Respir Med. 2020;8:895‐904. 32199466 10.1016/S2213-2600(20)30033-3

[38] Funazo T, Nomizo T, Kim YH. Liver metastasis is associated with poor progression‐free survival in patients with non‐small cell lung cancer treated with nivolumab. J Thorac Oncol. 2017;12:e140‐e141. 28838713 10.1016/j.jtho.2017.04.027

[39] Tournoy KG, Thomeer M, Germonpre P, et al. Does nivolumab for progressed metastatic lung cancer fulfill its promises? An efficacy and safety analysis in 20 general hospitals. Lung Cancer. 2018;115:49‐55. 29290261 10.1016/j.lungcan.2017.11.008

[40] Topalian SL, Hodi FS, Brahmer JR, et al. Five‐year survival and correlates among patients with advanced melanoma, renal cell carcinoma, or non‐small cell lung cancer treated with nivolumab. JAMA Oncol. 2019;5:1411‐1420. 31343665 10.1001/jamaoncol.2019.2187 PMC6659167

[41] Qin BD, Jiao XD, Liu J, et al. The effect of liver metastasis on efficacy of immunotherapy plus chemotherapy in advanced lung cancer. Crit Rev Oncol Hematol. 2020;147:102893. 32065969 10.1016/j.critrevonc.2020.102893

[42] Rebuzzi SE, Leonetti A, Tiseo M, et al. Advances in the prediction of long‐term effectiveness of immune checkpoint blockers for non‐small‐cell lung cancer. Immunotherapy. 2019;11:993‐1003. 31319742 10.2217/imt-2019-0107

[43] Rizvi NA, Hellmann MD, Snyder A, et al. Cancer immunology. Mutational landscape determines sensitivity to PD‐1 blockade in non‐small cell lung cancer. Science. 2015;348:124‐128. 25765070 10.1126/science.aaa1348 PMC4993154

[44] Hellmann MD, Ciuleanu TE, Pluzanski A, et al. Nivolumab plus Ipilimumab in lung cancer with a high tumor mutational burden. N Engl J Med. 2018;378:2093‐2104. 29658845 10.1056/NEJMoa1801946 PMC7193684

[45] Alexandrov LB, Nik‐Zainal S, Wedge DC, et al. Signatures of mutational processes in human cancer. Nature. 2013;500:415‐421. 23945592 10.1038/nature12477 PMC3776390

[46] Norum J, Nieder C. Tobacco smoking and cessation and PD‐L1 inhibitors in non‐small cell lung cancer (NSCLC): a review of the literature. ESMO Open. 2018;3:e000406. 30305940 10.1136/esmoopen-2018-000406 PMC6173248

[47] Wang GZ, Zhang L, Zhao XC, et al. The Aryl hydrocarbon receptor mediates tobacco‐induced PD‐L1 expression and is associated with response to immunotherapy. Nat Commun. 2019;10:1125. 30850589 10.1038/s41467-019-08887-7 PMC6408580

[48] Pirker R. Is smoking history the truly best biomarker for immune checkpoint inhibitor treatment in advanced non‐small cell lung cancer? ESMO Open. 2018;3:e000421. 30116594 10.1136/esmoopen-2018-000421 PMC6088342

[49] Abravan A, Faivre‐Finn C, Kennedy J, et al. Radiotherapy‐related lymphopenia affects overall survival in patients with lung cancer. J Thorac Oncol. 2020;15(10):1624‐1635. 32553694 10.1016/j.jtho.2020.06.008

[50] Campian JL, Ye X, Brock M, et al. Treatment‐related lymphopenia in patients with stage III non‐small‐cell lung cancer. Cancer Invest. 2013;31(3):183‐188. 23432821 10.3109/07357907.2013.767342 PMC4596242

[51] Tanizaki J, Haratani K, Hayashi H, et al. Peripheral blood biomarkers associated with clinical outcome in non–small cell lung cancer patients treated with nivolumab. J Thorac Oncol. 2018;13(1):97‐105. 29170120 10.1016/j.jtho.2017.10.030

[52] Bigot F, Castanon E, Baldini C, et al. Prospective validation of a prognostic score for patients in immunotherapy phase I trials: The Gustave Roussy Immune Score (GRIm‐Score). Eur J Cancer. 2017;84:212‐218. 28826074 10.1016/j.ejca.2017.07.027

