# Supplementary material for: Three models that predict the efficacy of immunotherapy in Chinese patients with advanced non‐small cell lung cancer
Source: Cancer Med. 2021 Aug 13;10(18):6291–303. doi: 10.1002/cam4.4171 (PMC8446565; doi:10.1002/cam4.4171)
Supplement: Supplementary file 1 — Table S1‐S2 [file CAM4-10-6291-s001.docx]

**Supplementary** **Table 1** Data used to calculate LIPI, mLIPI and EPSILoN scores

| Variables | LIPI | mLIPI | EPSILoN |
| --- | --- | --- | --- |
| ECOG PS |  |  |  |
| 0–1 |  |  |  |
| 2–3 |  | +1 | +1 |
| Smoking (packs/year) |  |  |  |
| <43 |  |  | +1 |
| ≥43 |  |  |  |
| Liver metastases |  |  |  |
| Yes |  |  | +1 |
| No |  |  |  |
| LDH (U/L) |  |  |  |
| ≤ULN |  |  |  |
| >ULN | +1 |  |  |
| ≤1.5*ULN |  |  |  |
| >1.5*ULN |  | +1 | +1 |
| NLR |  |  |  |
| <3 |  |  |  |
| ≥3 |  | +1 |  |
| <4 |  |  |  |
| ≥4 |  |  | +1 |
| dNLR |  |  |  |
| ≤3 |  |  |  |
| >3 | +1 |  |  |

LIPI, lung immune prognostic index; mLIPI, modified lung immune predictive index; EPSILoN, Eastern Cooperative Oncology Group performance status (ECOG PS), smoking, liver metastases, lactate dehydrogenase (LDH), neutrophil-to-lymphocyte ratio (NLR); ULN, upper limit of normal; dNLR, derived neutrophil-to-lymphocyte ratio.

**Supplementary Table 2** Prognostic groups identified by scores on the three predictive models

| Prognostic groups | LIPI | mLIPI | EPSILoN |
| --- | --- | --- | --- |
| Good (best) | 0 | 0 | 0 |
| Intermediate | 1 | 1 | 1–2 |
| Poor | 2 | 2 | 3–5 |
| Very poor |  | 3 |  |

LIPI, lung immune prognostic index; mLIPI, modified lung immune predictive index; EPSILoN, Eastern Cooperative Oncology Group performance status (ECOG PS), smoking, liver metastases, lactate dehydrogenase (LDH), neutrophil-to-lymphocyte ratio (NLR).
